# Hijacking the Message: Insights from Targeting mRNA Export and Translation in AML

**DOI:** 10.3390/ijms27209234

**Published:** 2026-10-17

**Authors:** Katherine L. B. Borden, Monica L. Guzman

**Affiliations:** 1 Department of Pharmacology, Northwestern University, Chicago, IL 60611, USA; 2 Robert H. Lurie Cancer Center, Northwestern University, Chicago, IL 60611, USA; 3 Division of Hematology/Medical Oncology, Department of Medicine, New York, NY 10021, USA; 4 Sandra & Edward Meyer Cancer Center, Weill Cornell Medicine, New York, NY 10021, USA; 5 Pharmacology Department, Weill Cornell Medicine, New York, NY 10021, USA

**Keywords:** eIF4E, CRM1/XPO1, mRNA export, mRNA translation, clinical trials

## Abstract

Cancer cells hijack RNA metabolism, including splicing, polyadenylation, nuclear export and translation, to change both the amount and the form of the proteins they produce. This creates therapeutic vulnerabilities beyond the mutations and translocations that have guided drug development in acute myeloid leukemia (AML), where outcomes remain poor. Using AML as a paradigm, this review focuses on two nodes of RNA metabolism: the eukaryotic translation initiation factor eIF4E, a cap-chaperone that escorts mRNAs through processing, export and translation, and CRM1/XPO1, the major nuclear exporter of proteins and select RNAs that partners with eIF4E for specific mRNA export activity. We describe the RNA biology dysregulated in patients, the mechanisms of the inhibitors that target these pathways, and their pre-clinical and clinical data across AML and other malignancies. Indeed, early-stage clinical trials in AML and other malignancies show promise, including the use of CRM1/XPO1 inhibitors (selinexor, eltanexor) and eIF4E inhibitors (including ribavirin and tomivosertib). We discuss how selecting patients by eIF4E status, confirming on-target activity, and designing rational combinations could improve next-generation therapies.

## 1. Scope of This Review

Clinical challenges continue to thwart improved outcomes in many heme malignancies, including acute myeloid leukemia (AML). For patients unfit for conventional induction therapy and without targetable mutations, the recommended treatment is hypomethylating agents (HMA) in combination with venetoclax; however, the outcomes for these patients are still dismal [1,2]. Azacytidine plus venetoclax provides remissions of ~18 months with poor responses to salvage therapies [3,4,5]. This suggests that new frontiers must be explored to improve these outcomes. Here we focus on mRNA biology, which is disrupted or hijacked in a variety of malignancies, including AML. A key step in gene expression is the role of mRNA processing, mRNA export and mRNA translation in determining both the final form of the protein and its levels. In this way, the instructions sent by the DNA can be subverted by cancer cells to underpin their necessary activities such as motility, survival, differentiation block and proliferation [6,7]. Modulation of the final form of the transcript and resultant protein can arise due to dysregulation of mRNA splicing or polyadenylation, while increased mRNA export can increase the amount of transcript available to the translation machinery without altering the levels of the RNA [6,7,8,9]. Similarly, increased translation efficiency (the number of ribosomes per transcript) increases the production of protein again without altering the levels of the transcript. Thus, modulation of splicing or polyadenylation can alter the form of transcripts, while increased RNA export or translation efficiency can increase protein levels [6,7,10]. In this way, factors that act in these processes and are dysregulated in malignancy represent important points to target in new therapies. Here, we will focus on the eukaryotic translation initiation factor eIF4E, a protein that acts in several steps in RNA processing, polyadenylation, nuclear mRNA export and translation, providing a unique leverage point to target multiple steps in dysregulated mRNA metabolism simultaneously [6,7,8,11,12,13,14]. We first describe the molecular activities of eIF4E, which are generally conserved across cancer cell types and then discuss the molecular basis for its targeting in cancer model systems focusing on AML and heme-malignancy, with the additional inclusion of solid tumor evidence given the conserved molecular mechanisms at play. Finally, we describe several early-stage clinical trials targeting eIF4E in patients. Early-stage clinical trials targeting other components of the translation machinery are also underway; although these are beyond the scope of this review, interested readers are encouraged to consult the growing literature in this area. Moreover, while we focus on increased translation efficiency in this review, translation dysregulation can manifest as impairment of selective translation, as in ribosomopathies and potentially in NPM-mutated AML [15,16,17]. We note that there are three eIF4E family members and this review focuses on eIF4E1, which we refer to here simply as eIF4E.

## 2. eIF4E in AML and Other Cancers

eIF4E is dysregulated in a wide variety of cancers, including hematological malignancies such as AML, lymphomas and multiple myeloma (MM) [8,9]. Its elevation is associated with poor outcomes [8,11]. In model systems, eIF4E overexpression promotes foci formation, growth in soft agar, apoptotic rescue, and motility and can lead to differentiation blocks in AML cells [18,19,20,21,22]. In xenograft mouse models, elevated eIF4E correlates with increased tumor numbers, invasion, and metastases [23,24,25]. It was shown that HoxA9, a transcription factor that drives a subset of AMLs, drives leukemogenesis in mouse models in part through its capacity to directly bind eIF4E and stimulate eIF4E-dependent RNA export [26,27,28]. Indeed, eIF4E plays an essential role in induction and maintenance of HoxA9-dependent leukemia [26]. In CDX models of AML, genetic reduction of eIF4E leads to delayed engraftment in the bone marrow and increased survival [29]. In transgenic models of eIF4E overexpression, mice develop a variety of cancers [30]. While eIF4E-mediated transformation was thought to rely only on increased translation of oncogenic transcripts [14], eIF4E’s nuclear functions are also critical to its oncogenic potential [13,31,32,33,34,35,36,37], as is nuclear import of eIF4E and its capacity to modify the nuclear pore [33,36]. eIF4E overexpression is positioned to modulate the nuclear export of thousands of mRNAs, including those involved in driving DLBCL such as *MYC* and *BCL2* [34], as well as in T-cell lymphomas, where there is increased export of mRNAs encoding the transcription factor GATA-3 and GATA-3-dependent targets and ribosome biogenesis factors [37]. Inhibiting this pathway in T-cell lymphomas also reprogrammed export in neighboring lymphoma-associated macrophages (LAMs) in the tumor microenvironment [11,37].

In patients, eIF4E levels are increased in many cancers, where it generally correlates with poor prognosis [8,19]. In a subset of AMLs, eIF4E protein levels are substantially elevated and eIF4E forms abnormally large nuclear bodies relative to those found in CD34+ bone marrow (BM) cells from healthy volunteers or to normal eIF4E AML [11,21,35,38,39]. High-eIF4E AML is enriched in French American British (FAB) M4/M5 AML subtypes as well as a fraction of M1/M2 AML subtypes (>150 specimens examined to date) [21,35,38,39]. While FAB subtypes are no longer used to classify AML, these data show that a substantial fraction of AML patients have elevated nuclear eIF4E protein, in addition to the expected cytoplasmic eIF4E. Nuclear enrichment of eIF4E correlated with elevated eIF4E-dependent RNA export relative to normal cells or normal-eIF4E AML specimens [21,35,38,39]. It is notable that CD34+ cells from healthy volunteers and AML cells both have nuclear and cytoplasmic eIF4E [11,21,40]. Survival analysis of a 609 AML patient cohort (Leucegene.ca) shows that AML patients with elevated *EIF4E* mRNA levels relative to the median had significantly reduced survival (*p* = 0.0438) [11]. Another study also showed that eIF4E mRNA levels are on average elevated in AML patients, particularly the M5 subgroups, relative to healthy volunteers (*p* < 0.001) [40]. Consistent with the Leucegene study, this latter work found a trend towards elevated *EIF4E* mRNA levels and poor outcomes in AML (*p* = 0.12; N = 230 patients) and suggested in their case that the inclusion of more patients would bring this to significance [40]. In all, eIF4E is dysregulated in a subset of primary cancer patients and is positioned as a therapeutic target in several malignancies [6,7].

## 3. Molecular Activities of eIF4E in the Nucleus and Cytoplasm

**Figure 1:**
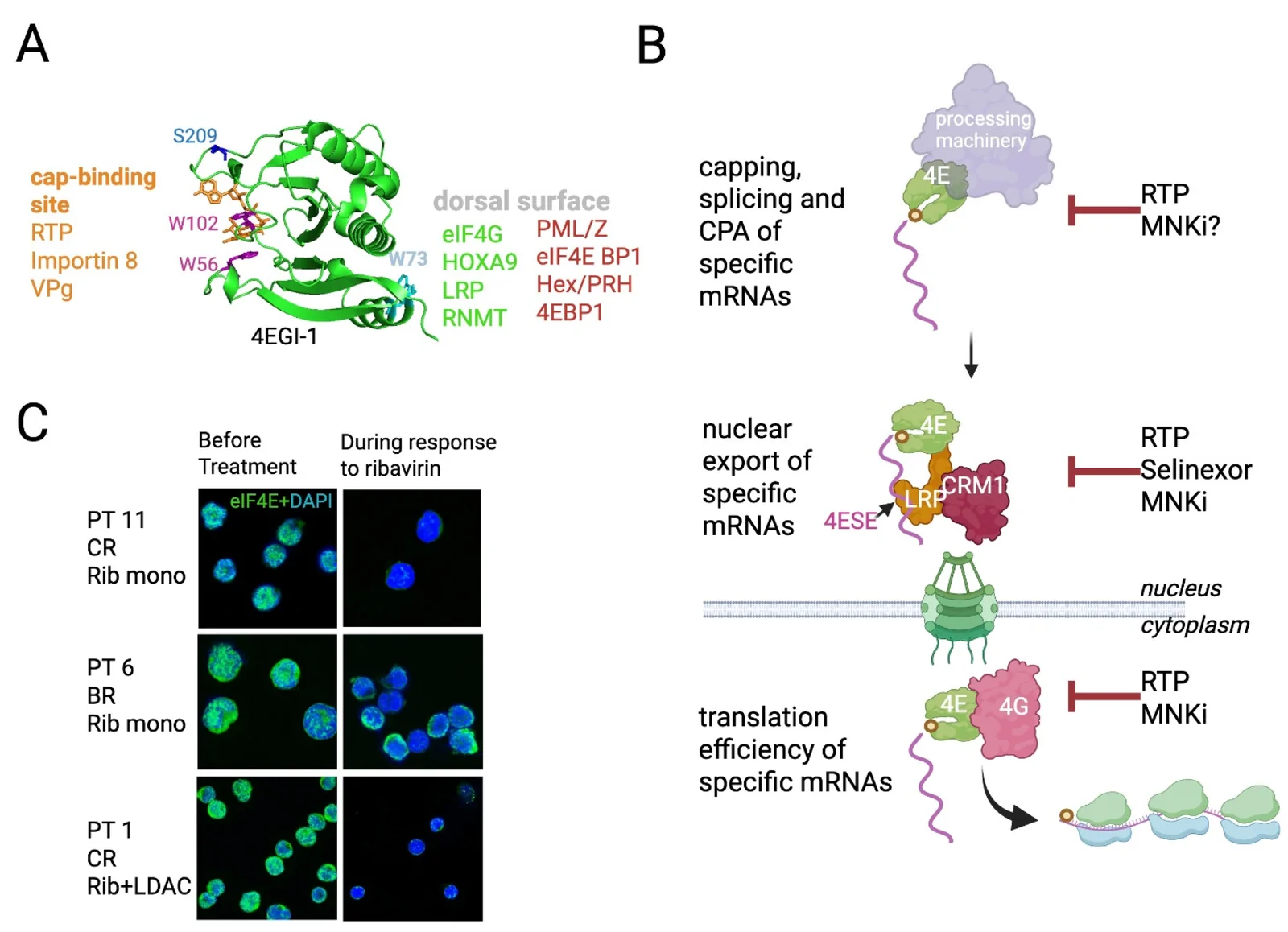
eIF4E structure, function and targeting in patients. (**A**) Human eIF4E structure and binding partners. Human eIF4E is shown complexed to the m^7^G cap, which is intercalated between W102 and W56 of eIF4E [50]. The structure was visualized in PYMOL (PDB code 1IPB [50]). The cap-binding site (orange) and dorsal surfaces, as indicated by W73 (gray), are indicated as binding partners and small molecules that bind to eIF4E. On the dorsal surface, negative eIF4E regulators are in red and positive regulators or factors required for eIF4E’s activities are in green. S209, the phosphorylated residue, is in dark blue. The commonly used laboratory inhibitor 4EGI1 is shown as well [51]. LRP refers to the leucine rich pentatricopeptide repeat protein (LPRPRC). Not all known binders are displayed for ease of viewing. A comprehensive list is provided in Mars et al. [8]. (**B**) The major processing steps that eIF4E is involved in as a cap-chaperone. The gray RNA processing protein complex indicates that while immunoprecipitation reveals physical association with the processing machinery, the exact biochemical complexes are not yet known. By contrast, these are known for the RNA export and translation activities of eIF4E. The beige ball denotes the m^7^G cap [8,9]. (**C**) Confocal micrographs from patient specimens demonstrating molecular response to eIF4E targeting by ribavirin, as observed by re-localization of eIF4E from the nucleus to the cytoplasm, corresponded to objective clinical improvement from patients from the ribavirin monotherapy trial who achieved a complete remissopm (CR) (patient 1), a blast response (BR) (patient 6) and a patient who achieved a CR in the ribavirin LDAC combination trial. eIF4E staining is in green and DAPI (blue) marks the nucleus [35,38]. Micrographs represent single sections through the plane of the cells and were collected before treatment and during responses, with the same magnification and displayed with the same digital zoom [35,38]. The figure was constructed in BioRender.

eIF4E, a factor conserved from plants to humans, is a 25 kDa protein that directly binds the 5′-7-methylguanosine (m^7^G) “cap” on the 5′ end of target transcripts using its evolutionarily conserved cap-binding pocket [8,9,41] (Figure 1A). There are over 80 proteins known to physically associate with eIF4E, with many of these interactions bridged by other proteins or RNA [8]. The majority of direct eIF4E partners reported to date interact via its dorsal surface, which is distal from the cap-binding site, but there is emerging evidence for binding of factors that can directly block mRNA interaction by associating with the cap-binding site [8,9] (Figure 1A). In cells, eIF4E is found in both the cytoplasm and nucleus in plants, yeast, flies, mice and humans [12,41,42,43,44] and thus is positioned to modulate a wide array of cap-dependent mRNA processing steps including capping, splicing, cleavage and polyadenylation as well as mRNA export and translation [8,9,11,13] (Figure 1B). Studies comparing eIF4E’s impacts on nuclear mRNA processing with the canonical cap-binding complex (CBC) suggest these factors influence the processing of discrete subsets of mRNAs [41]. In all, eIF4E impacts several aspects of transcript processing, potentially rewriting the message, with its best-described functions in the nuclear export and translation of specific transcripts, a means to amplify protein production in the absence of altering transcription or mRNA stability [8,9].

To conduct these multifactorial effects, eIF4E binds to the capped mRNA targets and the appropriate machinery necessary (Figure 1B) [7,8,9,11,13,45]. During eIF4E-dependent translation, eIF4G serves as a platform protein binding eIF4E-capped mRNA complexes and recruits other translation factors and the ribosome (Figure 1A,B). eIF4E overexpression increases translation efficiency (more ribosomes per transcript) of these target mRNAs. In the nucleus, the best characterized role of eIF4E is in nuclear export of select mRNAs; this in turn elevates cytoplasmic levels of transcripts and increases their availability to ribosomes without altering transcription [10,12,21,37,46]. In multiple cancer models, ~3000 transcripts are immunoprecipitated with eIF4E in the nucleus and many are likely targets of eIF4E-dependent RNA export, with these transcripts encoding proteins acting in malignancy and also in therapeutic resistance [14,34,46,47]. In this case, eIF4E employs the CRM1/XPO1 nuclear export receptor to engage the nuclear pore complex in order to transit RNA cargoes through the nuclear pore; by contrast, bulk mRNAs use the NXF1/NXT1 bulk RNA export receptor [33,34,42,45,48,49]. Additionally, eIF4E remodels the nuclear pore to promote export, which is associated with its oncogenic activity [33]. As discussed above, eIF4E also modulates splicing and indeed eIF4E is immunoprecipitated with the splicing machinery and pre-mRNA (splicing substrates) in a cap-dependent manner [11,48] as well as modulating the production of the splicing machinery [11]. Indeed, eIF4E elevation is associated with alternative splicing of ~4000 transcripts in primary high-eIF4E AML patients’ specimens relative to normal-eIF4E AML patients [11]. Finally, eIF4E was immunoprecipitated with the CPA machinery and modulated this process on model RNAs as well as for *CCND1* and *MCL1* [13]. In all, eIF4E drives protein production by increasing the cytoplasmic levels of specific RNAs (via export) and enhances translation efficiency of selected RNAs in the cytoplasm and is also positioned to modulate the form of the mRNA and thus the activity of resultant proteins *via* splicing and potentially CPA [8,9,11,48] (Figure 1B).

Importantly, mRNAs are sensitive to different levels of regulation by eIF4E based on their complement of cis-acting elements within their mRNAs, known as USER codes [8,9,49,52]. In demonstration of selectivity, note that targeting eIF4E genetically or pharmacologically only influences the translation of about 50% of global protein synthesis, with impacts on specific, not all, mRNAs such as *MYC*, nor does it impair all mRNA export or splicing. Indeed, in the nucleus, eIF4E binds to thousands of mRNAs, but not all; and of these, it influences the nuclear RNA export of a subset of transcripts and thousands, but not all, splicing events. In terms of specificity, eIF4E translation targets tend to have specific USER codes which sensitize transcripts to regulation by eIF4E. For translation, complex, highly structured 5′ untranslated regions (UTRs) are often associated with eIF4E sensitivity, as well as more defined sequences such as cytosine-enriched regulator of translation CERTs [8,9,53]. By contrast, RNA export targets have a ~50-nucleotide element in their 3′UTR denoted the eIF4E sensitivity element (4ESE) [1,10,12,21,54]. These USER codes are transferable to model mRNAs, leading to their sensitization of eIF4E-dependent mRNA export or translation in this case. Other USER codes will likely be discovered. Indeed, eIF4E does not impact all mRNAs to the same extent; this selectivity is highlighted by findings that housekeeping transcripts, e.g., *GAPDH*, are not RNA export, splicing or translation targets of eIF4E [11,12,19].

## 4. eIF4E and Viral Infections Associated with Cancers

Some cancers are associated with viral infections, particularly HPV, which drives cervical and oral cancers. Interestingly, eIF4E plays a functional role in HPV infection [55]. For example, ~98% of HNSCC patients have elevated eIF4E and p-eIF4E levels and similarly, the majority of cervical cancers are also characterized by elevated eIF4E [56,57]. HPV-mediated transformation occurs, in part, by targeting proteasomal degradation of p53 and pRB tumor suppressors through expression of the viral E6 and E7 oncoproteins [58]. In cell line models, the HPV E6 viral oncoprotein stimulates both eIF4E-dependent translation as well as increased levels of eIF4E itself through its enhanced transcription [57,58,59,60]. In this way, E6 plays a key role in hijacking the host cell translation machinery to favor translation of viral factors. E6 and E7 stimulate translation of capped mRNAs, including known eIF4E targets VEGF and C-MYC as well as E6 and E7 themselves [58]. While not a known driver of cancer, respiratory syncytial virus (RSV) infection is an important issue for cancer care since it is common in patients due to therapy-driven immunosuppression. RSV components recruit the host translation machinery and ribosomes for production of viral capped RNAs. During RSV infection, viral inclusion bodies are formed where viral mRNAs and the host translation machinery, including eIF4E, are concentrated, presumably to translate these viral factors [59]. Thus, eIF4E plays an important role in viral infections important to cancer and cancer care.

## 5. Strategies to Target eIF4E in the Lab and in the Clinic

There have been several modalities to target eIF4E in cell line and animal models, with a handful of these making it to clinical trials. We focus on the mechanism of action for the strategies that have been tested in patients. There are five main classes of drugs that have been used to target eIF4E in clinical trials: (1) cap competition using ribavirin; (2) selinexor, which targets CRM1/XPO1 and thus eIF4E-dependent nuclear mRNA export; (3) inhibitors of MNK1/MNK2, which reduce eIF4E phosphorylation; (4) antisense oligonucleotides to repress eIF4E production; and (5) signaling networks upstream of eIF4E (Table 1 and Table 2). We note that these modalities could elicit both eIF4E-dependent and independent impacts and the latter may improve efficacy or drive unwanted toxicity. These multi-targeting effects are also discussed. We first describe their mechanism of action and then their clinical efficacy for each of these strategies.

### 5.1. Cap Competitors to Target Multiple Functions of eIF4E Simultaneously

eIF4E relies on its m^7^G cap-binding activity to act in its nuclear activities, such as its capacity to act with the splicing machinery and to export mRNAs, as well as for its cytoplasmic activities in translation [8,9]. Indeed, eIF4E can be considered a cap-chaperone escorting client mRNAs through processing, export and translation steps [6,7,92,93]. Both its mRNA export and translation activities contribute to its oncogenic phenotype, while the relevance of its splicing, capping and CPA function to these phenotypes is not yet known [8,9]. Thus, cap competitors are positioned to influence all eIF4E’s functions simultaneously, including those related to its oncogenic transformation (Figure 1A,B). We focus on ribavirin as it is the first described cap-competitor and the only one tested in clinical trials (Table 1; Supplementary Table S1).

The antiviral drug ribavirin was identified serendipitously as a cap-competitor during studies into arenavirus infection, where ribavirin is the only treatment [94]. In terms of eIF4E function, ribavirin acts as an m^7^G-cap competitor, directly binding eIF4E, as shown by NMR, cap chromatography, fluorescence binding assays, and mass spectrometry [95,96,97]. To ensure that ribavirin binding was not restricted to contexts with purified proteins, eIF4E was shown to bind to ^3^H-ribavirin in intact cells [25,94,95]. Since the initial discovery of this activity, over 30 groups have demonstrated that ribavirin inhibits eIF4E’s activities in RNA export, translation, and oncogenic transformation and that ribavirin parallels genetic knockdown of eIF4E [25,26,27,41,97,98] (Supplementary Table S1). Moreover, eIF4E overexpression rescues cells from ribavirin treatment [99,100,101].

Interestingly, ribavirin binding to eIF4E impairs its capacity to bind Importin 8, the factor that imports eIF4E into the nucleus of AML and other cell types [36,102] (Figure 1A). Indeed, ribavirin causes redistribution of eIF4E to the cytoplasm, depleting the nuclear content both in patient specimens and in cell line models [35,38,60]. This is linked with impaired nuclear export of eIF4E-dependent mRNAs (since eIF4E cannot enter the nucleus, it cannot export the mRNAs) [36,102]. Thus, ribavirin treatment reduces the nuclear fraction of eIF4E in AML cells and in AML patients responding to ribavirin treatment, leading to a mainly cytoplasmic distribution which coincides with objective clinical response in patients as shown by monitoring AML specimens as a function of patient response (Figure 1C) [35,38,60]. This pattern of redistribution is key for monitoring molecular targeting of eIF4E in patients using confocal microscopy, which is ideal for situations where material is limited. In the cytoplasm, ribavirin interferes with eIF4E-dependent translation consistent with its physical association with the cap-binding site in lymphoma and solid tumor cells [34,35,36,38,96].

Recent studies showed that ribavirin synergizes with inhibitors of inosine monophosphate dehydrogenase (IMPDH), while early studies suggested that ribavirin directly inhibited IMPDH to impair GTP production [86,103]. These latter studies were based on the use of purified IMPDH proteins and addition of pure ribavirin monophosphate RMP [94]. However, RMP is a transitory intermediate on the path from ribavirin to ribavirin diphosphate (RDP)/ribavirin triphosphate (RTP), with little accumulation of RMP in cells, as seen by the ratio of RTP + RDP to RMP of 8:1. By contrast, eIF4E can bind all these ribavirin metabolites as well as ribavirin itself [94]. Some groups observed ribavirin-induced inhibition of IMPDH which was >25 times weaker than FDA-approved IMPDH inhibitors such as mycophenolic acid (MPA); other groups did not observe this inhibition [94]. For example, ribavirin inhibits the growth of mouse T-lymphoma cells by 50% at clinically relevant doses [94]. Consistent with acting independently of IMPDH, guanosine addition, which reverses the effects of IMPDH inhibitors, increased the cytotoxicity of ribavirin in these lymphoma cells [94]. Other IMPDH inhibitors such as tiazofurin failed to inhibit eIF4E-dependent mRNA export, eIF4E-dependent modulation of Akt phosphorylation or eIF4E-mediated anchorage-independent growth in cancer cells; at the same dose, ribavirin efficiently inhibited these eIF4E-dependent activities [94]. Tiazofurin impaired IMPDH activity, while ribavirin did not [94]. Interestingly, combinations of ribavirin and IMPDH inhibitors or genetic knockdown of IMPDH better inhibited cancer cell proliferation than either alone, were more effective in reducing experimental autoimmune encephalomyelitis and synergized to reduce viral load in RSV and human metapneumovirus infections [94,103,104]. Ribavirin does not appear to have major impacts on IMPDH in most cellular contexts but does have substantive impacts on eIF4E (Supplementary Table S1). Indeed, combinations of eIF4E inhibitors such as ribavirin and IMPDH inhibitors could be a therapeutically promising direction in cancer.

To date, there is no single defined antiviral mechanism for ribavirin [94]. Effects on IMPDH are considered irrelevant to its antiviral effects since more potent IMPDH inhibitors have little impact on most viral infections [94] and indeed ribavirin synergized with MPA to reduce viral infections [103]. The error catastrophe mechanism, where ribavirin is incorporated into viral RNA, is observed in laboratory conditions at concentrations of 1 mM ribavirin, which is not achievable in humans, with plasma levels typically ranging from 10 to 80 micromolar [35,38,60,94]. Global analysis of the transcriptome in human PBMCs indicates that ribavirin does not influence the transcriptome [94]. Importantly, no ribavirin is incorporated into RNA or DNA of human cells, nor does it repress DNA or RNA synthesis [94]. Interestingly, ribavirin can be misincorporated as the m^7^G cap of vaccinia virus mRNAs. By contrast, in human cells no radiolabeled ribavirin was incorporated into cellular mRNAs as the cap or within the RNA body [94]. Given that many viruses require eIF4E for infectivity and/or their life cycle, it seems likely that eIF4E plays a role in ribavirin-dependent effects in many viral infections. As an example, ribavirin, through its impacts on eIF4E, increased sensitivity to 5-fluorouracil in a HPV-driven cervical cancer mouse model, a standard of care in this malignancy [105].

As to the clinical impact of ribavirin in high-eIF4E AML, the majority of published trials targeting eIF4E (9/15) have used ribavirin and thus far have provided the largest number of objective clinical improvements (Table 1). The first trial to target eIF4E in any context in humans was in refractory or relapsed (R/R) AML patients with elevated eIF4E protein levels, with most patients receiving 1-2 prior rounds of therapy [35]. In this proof-of-principle multi-center trial, 5/11 patients treated with oral ribavirin presented with objective clinical responses including 1 complete remission (CR), 2 partial remissions (PR), 2 blast responses (BR; ≥50% drop in the number of leukemia cells in the bone marrow). In this study, there was a clear correlation between targeting eIF4E and clinical improvement. Molecular targeting included re-localization of eIF4E from the nuclei of high-eIF4E AML patients to the cytoplasm (Figure 1C), lowered mRNA export activity of eIF4E, lowered levels of downstream targets of eIF4E, including Cyclin D1 protein levels, reduced export of *NBS1* mRNA, and lowered phospho-AKT, consistent with studies demonstrating that eIF4E could act upstream of AKT using NBS1 [8]. Unexpectedly, lowered levels of eIF4E were observed, which were not observed in cell culture. This likely reflects that ribavirin selectively reduced the number of high-eIF4E AML cells in patients [35,38,60]. By contrast, there was no eIF4E targeting in patients that did not achieve an objective clinical improvement [6,29,64]. Thus, this was the first study to demonstrate that molecular targeting of eIF4E was possible and additionally that this corresponded to clinical responses. After these initial promising results, ribavirin was combined with low-dose cytarabine (LDAC) in another multicenter trial in high-eIF4E AML patients who had received, on average, 2–3 prior therapies [38]. Similar levels of responses were achieved as in the first trial, including 2 CRs, including a patient who responded for 24 months, 1 PR, 2 BR, with a total of 5/14 responses for patients who achieved the targeted ribavirin levels in their plasma. Responding patients also demonstrated a re-localization of eIF4E to the cytoplasm and, upon relapse, nuclear enrichment of eIF4E [38]. It was noted that LDAC impaired the oral absorption of ribavirin, suggesting other combinations would be more beneficial. In these first two trials, patients eventually relapsed and several of these manifested a novel form of drug resistance: inducible drug glucuronidation, which led to resistance not only to ribavirin but also to cytarabine and other drugs [38,95]. A proof-of-principle trial in third- and fourth-line high-eIF4E AML patients was the first study to target glucuronidation, which corresponded to ribavirin targeting of eIF4E and clinical response using similar assays to those above [60,95]. It is notable that the best responses were observed in patients in the ribavirin monotherapy trial who had received only 1–2 prior therapies on average and thus, not surprisingly, the number of prior therapies led to a reduced number of responses. In all, eIF4E targeting with ribavirin in high-eIF4E AML led to clinical benefit. Given these promising findings, there is a trial scheduled for Winter 2027 at Northwestern combining ribavirin with standard of care in de novo high-eIF4E AML patients. In all, targeting eIF4E through its relocalization, reduced mRNA export and reduced production of protein targets corresponded with objective clinical improvement in AML.

Many types of lymphoma are known to be characterized by elevated eIF4E. The impacts of targeting eIF4E in patients with ribavirin were also examined in R/R heavily pretreated indolent follicular and mantle cell lymphoma. In a retrospective study, the history of 10 lymphoma patients with active RSV infection was followed [61]. These patients received 10 days of ribavirin treatment, which effectively cleared the RSV infection but surprisingly also led to a dramatic reduction in tumor size by PET scan. 10/10 patients achieved a CR or PR. In a related case study by another group, a patient with heavily treated follicular lymphoma received a course of ribavirin and sofosbuvir for Hepatitis C, clearing both the infection and achieving a CR, which was maintained for over 1 year at last follow-up [64]. There was no molecular monitoring of eIF4E in these studies, but it is notable that eIF4E plays roles in both virus infections [106]. These findings suggest eIF4E-targeting in current lymphoma therapies could be clinically beneficial.

It is worth describing work in solid tumors as they provide additional examples. As described above, eIF4E plays a role in HPV viral infections. In previously untreated HPV-positive oral cancers, ribavirin was added to the standard of care carboplatin/paclitaxel, and it was found that its addition allowed reduction in chemotherapy doses, leading to the same remission rates but with less toxicity [62]. eIF4E was not measured in these patients. In a second trial, ribavirin was used in HPV-positive oral cancers as a monotherapy [63]. In this population, there were, on average. 3 prior rounds of chemotherapy as well as radiotherapy. Here, in an arm to monitor molecular responses only, 4/6 patients showed 2–3-fold reduced p-eIF4E levels after 14 days of ribavirin treatment, indicative of targeting eIF4E. In the therapeutic arm, the best response was 222 days and 173 days of stable disease out of nine patients. No measurement of eIF4E activity was conducted in the therapeutic arm. Thus, it is unknown whether, in these heavily pretreated patients with HPV-related cancer, eIF4E-phosphorylation reduction persisted beyond 14 days or indeed occurred in patients on the therapeutic arm of the study. In addition, ribavirin has been used to target eIF4E in castration-resistant prostate cancer, where the best responses were 2/5 SDs [65]. In these latter two studies, it appears that targeting eIF4E alone may not be sufficient to achieve a response in heavily pretreated patients.

### 5.2. Selinexor Targets Xpo1/crm1 and Eif4e-Dependent RNA Metabolism

CRM1, also called exportin-1 or XPO1, is the principal nuclear export receptor for more than 200 cargo proteins [107,108]. Among them are essentially every major tumor suppressor and growth regulator (e.g., p53, p73, p21, p27, FOXO and NPM1) [107,108]. CRM1/XPO1 directly engages the nuclear pore complex (NPC) to transit cargoes to the cytoplasm [109]. At steady state, CRM1 is almost entirely nuclear, indicative of rapid recycling of CRM1 to the nucleus after cargo release. CRM1 is the major nuclear exporter of proteins in the cell and uses protein adaptors, such as PHAX, NMD3 and LRPPRC, to traffic UsnRNA, rRNA and a select group of eIF4E-dependent transcripts from the nucleus, respectively [109]. CRM1 elevation is generally associated with increased metastases in solid tumor patients and poor prognosis in AML [110]. Selinexor, a CRM1 inhibitor, is FDA-approved for refractory/relapsed (R/R) multiple myeloma (MM) and diffuse large B-cell lymphoma (DLBCL) [109], tumor types also associated with elevated eIF4E [34,111,112,113]. Selinexor covalently binds to the cargo-binding site in CRM1 [109]. Selinexor activity is attributed, in part, to its inhibition of the CRM1/eIF4E-dependent mRNA nuclear export pathway [37,46,112,114], an observation based on the earlier discovery that eIF4E-dependent mRNA export was CRM1-mediated [31]. Selinexor demonstrates substantial anti-AML activity while sparing normal cells in vitro [115]. Despite its role as a CRM1 inhibitor, selinexor does not elicit global impacts on the cellular proteome but rather targets factors associated with oncogenesis and chemoresistance [46]. CRM1/XPO1 was shown to be a therapeutic vulnerability in T-cell lymphoma independent of impacts on TP53 and PTEN [37]. The nuclear export of the mRNAs encoding the transcription factor GATA-3, GATA-3-dependent targets and ribosome biogenesis was dependent on both eIF4E and CRM1/XPO1 [37]. Selinexor inhibited this export pathway and, additionally, inhibited export in neighboring lymphoma-associated macrophages (LAMS) in the tumor microenvironment [37]. In terms of the interrelationship between eIF4E and CRM1/XPO1, eIF4E knockdown was shown to sensitize cells to selinexor, while eIF4E overexpression partially rescued these effects in MM models [112] further substantiating their functional interplay. Another example comes from DLBCL, where CRM1/XPO1 overexpression increased tolerance to genotoxic stress and here CRM1/XPO1 ferried eIF4E, the nuclear export adaptor THOC4, and mRNAs encoding DNA-damaging factors to the cytoplasm. Selinexor treatment reduced the capacity of lymphoma cells to repair DNA damage and thereby increased cell toxicity [46]. In a phase I clinical trial with selinexor with second-line chemoimmunotherapy in patients with R/R aggressive B-cell lymphomas, there were early promising signals. Of the 21 patients who received treatment, there were 7 CRs, 8 PRs and 3 SDs [46]. Samples were not available to monitor molecular markers.

In all, targeting CRM1/XPO1 demonstrated substantial promise in cell and animal models, providing the impetus for other clinical trials. For this discussion, we focus on AML, where Selinexor has demonstrated some activity as a single agent and in combination in clinical trials for AML (Table 2). The phase 1 monotherapy studies demonstrated a 14% objective response rate, with about a third of patients showing a ≥50% blast reduction, and responders lived markedly longer than non-responders (9.7 vs. 2.7 months) [74]. Later, a randomized trial (SOPRA) did not perform better than physician’s choice [75]. The median overall survival was 3.2 months for selinexor versus 5.6 months for physician’s choice. Adding selinexor to chemotherapy produced response rates of 40 to 70% in relapsed or refractory disease. It was used as a bridge to transplant where ~ 30–60% of patients reached allogeneic bone marrow transplant. However, almost none of these trials show superior results when compared with historical controls [1]. Unfortunately, a randomized study (HOVON/SAKK) showed that adding selinexor to 7 + 3 in fit elderly patients lowered CR/CRi from 80% to 59%, decreased 18-month overall survival (33% vs. 58%), and doubled 60-day mortality [81]. Most of these studies showed toxicity concerns, where nausea, anorexia, weight loss, fatigue and hyponatremia were observed at high rates in all trials (Table 2). Much of the central toxicity is attributed to CNS penetration, which led to the development of the second-generation compound eltanexor [86,88]. There is still promise for this better-tolerated second-generation compound, with a rationale for combinatorial therapies [86,88], and, importantly, the potential for stratification of the molecular subgroups such as splicing-factor-mutants, NPM1-mutant and NUP214-rearranged disease in which the dependency on nuclear export has been shown to be the greatest [116,117,118].

### 5.3. Targeting eIF4E Phosphorylation with Mnk Inhibitors

Increased levels of eIF4E as well as its phosphorylation at S209 are associated with aggressive cancers and linked to its oncogenic potential in model systems [18,30,99,100]. eIF4E phosphorylation increased eIF4E-dependent mRNA export by about 2-fold, increasing cytoplasmic availability of candidate transcripts to the ribosome without altering total mRNA levels of these targets [119]. Studies into eIF4E translation efficiency provide more controversial results, with some reports suggesting eIF4E translation efficiency is phosphorylation-dependent and others that it is not [120,121]. Likely, the effects of phosphorylation could be transcript- and context-specific. MAPK-interacting kinases MNK1 and MNK2 are both capable of eIF4E phosphorylation and appear to be the only kinases to do this [121]. MNK1/2 knockout mice do not have phosphorylated eIF4E or a phenotype, thus positioning these kinases as therapeutic targets [121]. It is important to note that many proteins are targets of MNK1/2, including hnRNPA1, the polypyrimidine tract-binding protein-associated splicing factor (PSF), Sprouty 2 and cytosolic phospholipase A2, as well as others [122]. Thus, impacts of MNK inhibitors need to be considered within a multifactorial lens. There has been a wide array of MNK inhibitors, but we will focus here on eFT508, also known as tomivosertib, given that this is the only MNK inhibitor used in clinical trials (Table 1). Other MNK inhibitors are in development [123,124]. It is of interest that ribavirin also reduced eIF4E phosphorylation in ALL, ovarian and breast cancer cells [25,125,126], and ribavirin monotherapy reduced eIF4E phosphorylation in patients with oral HPV cancers [63] (Table 1). The mechanism for this reduction is unknown.

Tomivosertib is a dual MNK1/2 inhibitor that has shown substantial anti-neoplastic responses in solid tumors, lymphomas and leukemias [127,128]. The development of tomivosertib represented an important new tool to study the relevance of eIF4E phosphorylation and phosphorylation of other MNK targets. Tomivosertib reduced cell viability and leukemic progenitor colony formation assays in a dose-dependent manner [127]. The authors show that a combination of tomivosertib with venetoclax led to even greater impacts on leukemic cell proliferation. Interestingly, venetoclax resistance can arise due to upregulation of MCL1, a target of eIF4E-dependent mRNA export and translation. In this study, AML FAB subtype M5 and FLT3 mutant cells are more sensitive to tomivosertib, with each agent reducing colony formation by 2-fold and the combination by 4-fold [127]. Tomivosertib has also had striking effects in solid tumors. Interestingly, translation of programmed death ligand 1 (PD-L1) is upregulated in MYC^Tg^/KRAS^G12D^ mice and this was reversed by inhibiting eIF4E phosphorylation with tomivosertib, which also reversed the aggressive neoplastic features of these tumors [129]. It will be interesting to examine the efficacy of tomivosertib in patients with acquired resistance to PD-1 blockade [129]. MNK pathways may manifest a rescue pathway from stress and thus targeting these in the context of chemotherapy-induced stress may lead to better outcomes [127,130,131].

To date, four clinical trials have examined the activity of tomivosertib in solid tumors (Table 1). All trials were in the heavily pretreated setting, and no studies of tomivosertib monotherapy have been reported. In metastatic breast cancer patients, paclitaxel was combined with tomivosertib, which corresponded to a reduction in phosphorylation of eIF4E [69]. It is unknown whether this is due to reduced eIF4E protein levels or specific reduction in phosphorylation. This breast cancer study is the only published study to monitor eIF4E phosphorylation in patients receiving MNK inhibitors. Of 19 patients, the best clinical response achieved was 3 cases of stable disease. In another trial combining tomivosertib with checkpoint inhibitor therapy in solid tumor patients who had failed prior checkpoint inhibitor therapy, 3 PRs were achieved in 39 patients [66]. Moreover, 7 NSCLC patients were progression-free for 24 weeks. This led to trials with tomivosertib and pembrolizumab in NSCLC patients, but there was no objective improvement in this patient group and similarly for prostate cancer (NCT04622007 and NCT03690141). In all, inhibitors of eIF4E phosphorylation may be more effective in less heavily pretreated populations, and it would be valuable to assess if eIF4E phosphorylation was targeted in these latter trials. Unlike ribavirin treatment, where molecular responses were restricted to patients who demonstrated clinical improvement in AML, it appears in metastatic breast cancer that tomivosertib decreased eIF4E phosphorylation, but this reduction did not correspond to objective clinical improvement. It may be that while eIF4E phosphorylation potentiates eIF4E activity, ribavirin more efficiently impairs eIF4E activity through cap-competition, leading to improved responses.

### 5.4. Genetic Repression of eIF4E with Antisense Oligonucleotides

Early studies in prostate cancer mouse models demonstrated impressive targeting of eIF4E with antisense oligonucleotides, which corresponded to reduced cancer burden in these animals [132]. There have been two published trials in this area. In 2011, a stage 4 solid tumor population was assessed [70]. There was a qualitative assessment of eIF4E levels suggesting its levels went down modestly with ASO treatment alone. Of these patients, 7/22 achieved SD as the best response, with no patients on therapy for more than 3 months [70]. In a follow-up trial using a next-generation ASO to eIF4E in combination with irinotecan, there were no objective improvements, with 7/15 patients achieving an SD [71]. While the ASO was shown to enter the tissue, no reduction in eIF4E protein levels was observed. In contrast to mouse models, it seems that the ASO did not reduce eIF4E in patients, consistent with the lack of objective responses. Studies examining mRNA vaccine strategies may be a valuable future direction for this modality.

### 5.5. Targeting Upstream Signaling to Inhibit eIF4E

Given that eIF4E is downstream of PKC and mTOR signaling, this strategy has been used in heavily pretreated patient populations. These treatments are multitargeting, i.e., not only influencing eIF4E but many other pathways as well, which may provide additional clinical benefit. Sapanisertib, known to decrease p-4EBP1 levels, led to 1 CR and 7 PR in 67 patients, which was deemed insufficient to continue its development in this indication [71] (Table 1). Note that p-4EBP1 is the form of the 4EBP1 inhibitor that does not bind to eIF4E and thus does not suppress eIF4E function. Whether decreased p-4EBP1 corresponded to a reduction in total 4EBP1 levels was not investigated. This would be important to establish since if 4EBP1 levels themselves were reduced, this could lead to a potential de-repression of eIF4E. The drug enzastaurin inhibits PKC and is also associated with increased eIF4E-4EBP1 complex formation, which should reduce the translation activity of eIF4E [71]. The combination of enzastaurin with R-CHOP in over 100 lymphoma patients was compared to R-CHOP alone (Table 1). In this case, there was no improvement [71]. Given that there is a plethora of protein factors that repress or stimulate eIF4E activity [8], it may be that targeting 4EBP1 simply leads to activation of compensatory networks. In such a case, determination of these networks could reveal new drug targets to use in combination with these strategies. Targeting 4EBP1 will only influence the cytoplasmic functions of eIF4E and thus strategies to target its nuclear functions may be an effective combination strategy.

## 6. Considerations to Build Better Next Generation Therapies to Target High-eIF4E Cancers

While there are promising clinical signals for some eIF4E targeted therapies, several challenges remain for their development. Some of these are relevant across cancer biology, such as that patient populations in trials are typically heavily pre-treated prior to exposure and already manifest multi-drug resistance mechanisms. For this reason, it is critical in new trials targeting eIF4E to: 1. monitor eIF4E status at the start of treatment to assess if high-eIF4E patients are more sensitive to eIF4E-targeted therapies as would be predicted by cell line and mouse models; 2. assess if the therapy targeted eIF4E and if this corresponded to patient response and 3. examine if patient dosing is reaching levels that make point 2 possible. With this in mind, patient selection for trials should be considered, i.e., screening patients for elevated eIF4E and examining whether drugs indeed enter the AML cells prior to starting therapies to select for the patients most likely to benefit from a given regimen. Standing in the way of success are a plethora of pharmacological drug resistance mechanisms as well as genetic compensation. The vast majority of cancer therapies require combinations and a better understanding of the compensatory networks in high-eIF4E cancer will likely be key to providing durable outcomes for these patients. Aside from selecting patients who can take up the relevant drugs, drug resistance could potentially be targeted in combination with eIF4E inhibitors. For example, next-generation glucuronidation inhibitors may serve to assist in this. Studies combining eIF4E and CRM1/XPO1 targeting will be interesting and may allow reducing selinexor/eltanexor doses and the associated toxicity in patients. In all, multiple strategies have validated that eIF4E dysregulation, and its associated modulation of transcript processing, export and translation, can be targeted safely in humans, with some strategies benefiting a subset of patients. The next challenge will be to make these outcomes durable and applicable to broader patient populations.

## Figures and Tables

**Table 1:** Clinical Trials Targeting the eIF4E/Cap-Dependent Translation Axis. Includes agents directly targeting eIF4E (ribavirin, antisense oligonucleotide. phosphorylation) and agents acting on upstream/downstream nodes of the cap-dependent translation pathway (MNK, mTOR, PKC inhibitors).

Regimen	Indication/Population	eIF4E-Pathway Biomarker Effect	Efficacy Outcome	Trial ID & Reference
**Ribavirin**				
**Ribavirin monotherapy**	**R/R AML with high eIF4E protein levels** *Patients had ~1–2 prior treatment* *N = 11*	In responding patients eIF4E re-relocalization from the nucleus to the cytoplasm, lowered eIF4E protein levels and lowered eIF4E proteins targets were observed. Patients with no response showed no targeting of eIF4E.	5/11 evaluable pts had objective clinical improvement: **1 CR, 2 PR, 2 BR, 4 SD, 2 PD**	**[35]** **NCT00559091**
**Ribavirin + low-dose** **Ara-C**	**R/R AML with high eIF4E protein levels** *Patients had ~ 2–3 prior treatments* *N = 14*	In responding patients eIF4E relocalization from the nucleus to the cytoplasm, lowered eIF4E protein levels and lowered eIF4E proteins targets were observed. Patients with no response showed no targeting of eIF4E.	*n* = 14 evaluable who reached >20 µM ribavirin (max dose), 5/14 had clinical improvement Among those**: 2 CR (24 m and 6 m), 1 PR, 2 BR, 2 SD (26%→14% blast drop)** *Ara-C interfered with oral absorption of ribavirin* *And had to be reduced to increase ribavirin to therapeutic levels*	**[38]** **NCT01056523**
**Ribavirin + Decitabine + Vismodegib**	**R/R AML with high eIF4E protein levels** *Patients had ~ 3–4 prior treatments* *n-10*	In responding patients eIF4E relocalization from the nucleus to the cytoplasm, lowered eIF4E protein levels and lowered eIF4E proteins targets were observed. Patients with no response showed no targeting of eIF4E.	**1 PR, 3 BR, 6 SD** (2 for 4–6 months) SD of 10 evaluable patients in the VDR arm	**[60]** **NCT02073838**
**10-day ribavirin + standard of care**	**R/R indolent follicular lymphoma & mantle cell lymphoma** *Heavily pretreated* *Retrospective, n-10* *DLBCL characterized by dysregulated eiF4E driving MYC, BCL2 and BCL6*	ND	**10/10 patients had CR or PR** Median OS 21.5 months (range 6.8–88.8) *Patients with lymphoma + RSV infection treated with ribavirin for RSV had dramatic tumor size reduction seen by PET responses*	**[61]** **NCT03585725**
**Induction chemo: Afatinib + Ribavirin + weekly Carboplatin/Paclitaxel**	**HPV+ HNSCC (oropharynx, stage IVA/IVB)** *Rationale: HPV16 E6 increases eIF4E tra* *nscription and cap-dependent translation; rib* *avirin showed anticancer activity in eIF4E-dependent HNSCC xenografts* *Previously untreated patients*	ND	**PR: 6 (67%); SD: 2 (22%); PD: 1 (11%)** 8/10 evaluable for follow-up; median follow-up 34.8 months Of 7 with induction chemo + post-treatment imaging: median DFS 2.5 yr, 86% survival Could lower levels of chemotherapy and get same response rate with ribavirin addition	**[62]** **NCT01721525**
**Ribavirin monotherapy**	**Recurrent/metastatic HPV related cancer with elevated p-eIF4E** *PK arm: 6 pts with high p-eIF4E (14-day treatment);* *Therapeutic arm: 12 total* *All pts heavily pretreated (avg. 3 prior chemotherapies + radiotherapy)*	PD arm: 4/6 patients had 2–3-fold reduction in p-eIF4E after 14 days PK p-Therapeutic arm: eIF4E not measured for therapeutic arm *Total eIF4E levels not measured—unclear if effect is phospho-specific or reflects lower total eIF4E*	Therapeutic arm**: 5/9 SD, 4/9 PD** 2 long-term SD (222 days, 173 days) PD arm: No evaluation for response given only treated for 14 days	**[63]** **NCT02308241,** **NCT01268579**
**Ribavirin + Sofosbuvir**	**Advanced Follicular Lymphoma with concurrent RSV infection** *n =* 1	ND	**CR continued more than 1 year at follow up** **Patient cleared RSV infection and achieved CR by CT scan**	**[64]**
**Ribavirin + Docetaxel (Phase 1/2a)**	**Castration-resistant prostate cancer** *n =* 5	ND	**PSA reduction in 2/5 patients; 3/5 stable disease**	**[65]**
**MNK Inhibitor (eFT-508/tomivosertib)**				
**eFT-508 (tomivosertib) added to continued checkpoint-inhibitor therapy**	**Insufficient response (SD >12 week or progression) to any FDA-approved checkpoint inhibitor in solid tumours** *Median 2 prior therapies* *n* = 39	ND	**3 PR (by RECIST) and 7 (41%) NSCLC patients were** progression-free at 24 weeks from 39 patients	**[66]** **NCT03616834**
**eFT-508 (tomivosertib) + pembrolizumab**	**NSCLC, PD-(L)1-positive: first line, or progressing on single-agent PD-(L)1** *n =* 68	**ND**	**No responses** Median PFS 13.0 week (combination) vs. 11.7 week (with only pembrolizumab + placebo) ***Development discontinued with no remissions***	**[67]** **NCT04622007**
**eFT-508 + standard of care**	**Castration resistant Prostate cancer**	ND	**16 enrolled** **, No patients completed the study**	**[68]** **NCT03690141**
**eF-208 + paclitaxel**	**Metastatic Breast Cancer** *n =* 19	**Substantial targeting of phospho-eIF4E** *Total eIF4E levels not measured—unclear if effect is phospho-specific or reflects lower total eIF4E* *Excellent targeting of eIF4E phosphorylation but little impact on tumour growth*	**Best response 3 SD (13 months, 2 × 8 months)** **19 patients**	**[69]** **NCT04261218**
**Antisense Oligonucleotides**				
**eIF4E ASO LYS2275796 monotherapy**	**Stage 4 solid cancers** *n = 22 evaluable*	eIF4E protein levels modestly lowered but were not quantified; lowered in both nuclei and cytoplasm	**No tumour response was observed** **7 pts: achieved SD for ~6 weeks and 15 PD**; none remained on therapy >3 months	**[70]** **NCT00903708**
**eIF4E ASO (ISIS 183750)+ Irinotecan**	**Advanced solid tumors/colorectal cancer (all R/R)** *n =* 19/24	No change in eIF4E protein levels in tumor 13/19 had reduced eIF4E RNA; protein unchanged (higher in some tumors after treatment) ASO confirmed to enter tumor tissue	**No objective tumour response was observed** **7/15 SD (6 stable at 16 weeks); no objective response** Median PFS: 1.9 months Dose level 1: 3/6 SD, 3/6 PD (no remissions) Dose level 2: 4/9 SD, 5/9 PD (no remissions)	**[71]** **NCT01675128**
**mTOR Inhibitors**				
Sapanisertib	**Advanced solid tumours who failed standard of care** *n =* 64	Significant reduction in 4E-BP1 levels observed in some patients’ specimens before versus treatment matched biopsies	**1 CR and 7 PR** from 64 patients	**[72]** **NCT01058707**
**PKC Inhibitor**				
**Enzastaurin + R-CHOP vs. R-CHOP alone**	**Lymphoma**	Besides inhibiting PKC, enzastaurin increases eIF4E:4E-BP1 binding and reduces eIF4F complex formation (Dumstorf et al., Mol Cancer Ther 2010) **[73]**	No apparent improvement over R-CHOP alone With >100 patients	**[73]** **NCT03263026**

CR = complete remission. PR partial remission, BR blast response, SD stable disease, PD progressive disease. R/R refractory/relapsed.

**Table 2:** CRM1/XPO1 inhibitor trial results in AML.

Trial	Design & Population	Regimen	Key Efficacy	Safety/Notes	Reference	Trial Type
**NCT01607892**	Phase 1, dose-escalation, single-arm 95 patients with R/R AML	Selinexor monotherapy 16–70 mg/m^2^, once, twice or 3 times per week	ORR 14% (11 of 81 evaluable: 5 CR, 2CRi, 1 MLFS, 3PR). 31% (20 of 65) achieved ≥50% marrow blast reduction. Median OS 2.7 months; 9.7 months in responders.	No formal DLT; RP2D 60 mg twice weekly. Mostly grade 1–2 GI and constitutional events; fatigue the main grade ≥3 non-hematologic AE	[74]	Selinexor monotherapy
**NCT02088541** **SOPRA Study**	Phase 2, randomized 2:1, open-label ≥60 years, R/R AML inelegible for intensive chemothrapy/SCT (118 vs. 57)	Selinexor 60 mg twice weekly vs. investigator’s choice of therapy	Median OS 3.2 vs. 5.6 months (HR 1.18, 95% CI 0.79–1.75; *p* = 0.422) CR/CRi low in both arms (11.9% vs. 3.5%, *p* = 0.084). OOR 13.6% vs. 8.8%. No survival benefit in an unselected population.	Most common grade ≥3 Aes with Selinexor: thrombocytopenia, febrile neutropenia, anemia and hyponatremia. SAEs 73–82% (selinexor) vs. 66% (physician’s choice).	[75]	Selinexor monotherapy
**NCT02249091** **SAIL**	Phase 2 R/R AML (*n* = 42)	Selinexor + 7 + 3 (cytarabine, idarubicin); 40 mg/m^2^ BIW × 4 wk, amended to 60 mg flat BIW × 3 wk	ORR (CR/CRi/MLFS) 50%; Approx. 1/3 proceeded to allo-HSCT Median EFS 4.9, RFS 17.7, OS 8.2 months.	Original schedule: febrile neutropenia 85%, grade 3–4 diarrhea 56%. Amended dose improved tolerability	[76,77]	Selinexor + intensive chemotherapy
**NCT02299518**	Phase 1, dose-escalation Adults <60 y, R/R AML (*n* = 23)	Selinexor 30–55 mg/m^2^ BIW (d1, 3, 8, 10, 15) + MEC	ORR 43% (CR 26%, CRi 9%, MLFS 9%); CR/CRi 35%. 7/10 responders bridged to allo-HSCT. Median OS 8.5 months.	MTD/RP2D 30 mg/m^2^; DLT grade 3–4 hyponatremia. Febrile neutropenia, catheter infection, diarrhea, sepsis	[78]	Selinexor + intensive chemotherapy
**NCT02573363**	Phase 1 selinexor in combination with high dose cytarabine and mitoxantrone. Newly diagnosed or R/R high-risk AML (*n* = 20)	Selinexor 60 → 80 mg BIW + high-dose cytarabine/mitoxantrone (age-adjusted)	ORR 70% (CR 50%, CRi 15%, PR 5%). 57% of responders proceeded to allo-HSCT.	No DLT; RP2D 80 mg BIW. Febrile neutropenia 70%, diarrhea 40%, anorexia 30%, electrolyte abnormalities 30%	**[79]**	Selinexor + intensive chemotherapy
**NCT02403310**	Phase 1, single arm combined with cytarabine and daunorubicin. Newly diagnosed poor-risk AML (*n* = 21; median age 69)	Selinexor 60 or 80 mg BIW + daunorubicin and cytarabine (7 + 3); RP2D 80 mg BIW	CR/CRi in 10 of 19 evaluable (53%; CR 8, CRi 2); MTD not reached. Median OS 10.3 months; 38% alive at 28.9 months’ follow-up.	Grade 3–4 non-hematologic: febrile neutropenia 67%, diarrhea 29%, hyponatremia 29%, sepsis 14%	[80]	Selinexor + intensive chemotherapy
**NTR5902 /** **NL5748**	Randomized phase 2, open label ≥66 y (median 69) fit for intensive therapy (*n* = 102; 51 per arm)	Standard 7+ 3 induction with or without selinexor	CR/CRi 59% (selinexor) vs. 80% (control). 18-month EFS 26% vs. 45%; OS 33% vs. 58% No improvement in MRD negativity.	More infection, sepsis and treatment-related mortality. Addition of selinexor to standard chemotherapy negatively affected the outcome of elderly AML patients.	[81]	Selinexor + intensive chemotherapy
**NCT02416908**	Phase 1/2, single arm Adults with R/R AML (*n* = 40)	Selinexor 60 mg (d1, 5, 10, 12) + CLAG (cladribine, cytarabine, G-CSF)	CR/CRi 45% (18 of 40) vs. 33% historical (*p* = 0.16). Phase 2 endpoint not met, median EFS 6.1, OS 7.8 months; ~60% to allo-HSCT	30- and 60-day mortality 2.5% and 7.5%. Median recovery 28 d (ANC) and 38 d (platelets)	[82]	Selinexor + intensive chemotherapy
**NCT03661515** **PETHEMA phase I**	Phase 1, dose-escalation Younger adults with R/R AML (*n* = 14)	Selinexor 60, 80 or 100 mg weekly + FLAG-Ida	CR/CRi in 5 of 12 evaluable (42%); 4 patients (33%) proceeded to allo-HSCT. Median OS 6.0 and EFS 1.1 months; at 100 mg weekly, CR/CRi 66.7% and OS 13.6 months. No dose-limiting toxicities reported.	Grade ≥3 non-hematologic AEs in 78.6%; 3 of 14 patients (21.4%) had fatal adverse events	[83]	Selinexor + intensive chemotherapy
**NCT02093403**	Phase 1, dose-escalation R/R AML, or ≥60 y untreated and unfit (*n* = 25)	Decitabine 20 mg/m^2^ d1–10 + selinexor BIW (23–55 mg/m^2^); Recommended phase 2 dose: 60 mg flat BIW	ORR 40% (CR/CRi/MLFS). Responses in both newly diagnosed elderly and R/R cohorts	No protocol-defined DLT. Grade ≥3 hyponatremia 68%, febrile neutropenia, sepsis, hypophosphatasemia, pneumonia	[84]	Selinexor + low-intensity and targeted therapy
**NCT02530476**	Phase Ib/II clinical study of selinexor in combination with sorafenib in relapsed/refractory patients. FLT3-mutated R/R AML, including prior FLT3-inhibitor failure (n = 14 reported)	Selinexor 40, 60, or 80 mg BIW (3 wk on/ 1 wk off) + sorafenib 400 mg BID. Responses at 60 and 80 mg.	CRp/CRi in 4/14 (29%), 2 PR with >50% blast reduction (6/14). refractory patients (median 3 prior therapies); composite CR 35.7% Molecular CRs in ITD (2) and ITD+D835 (1) mutants.	Preclinical studies showed Selinexor upregulated phospho-FLT3, giving synergy with FLT3 inhibition and myeloid differentiation	[85]	Selinexor + low-intensity and targeted therapy
**NCT03955783**	Phase Ib, open-label. Adults with R/R AML (*n* = 19)	Selinexor 80 mg weekly + venetoclax 400 mg/day after ramp-up (RP2D)	ORR 21%; 2 patients (11%) achieved CR, DoR 7 and 9.1 months Median EFS 2.4, OS 6.4 months.	Grade 3–5: anemia 39%, neutropenia 33%, febrile neutropenia 28%, thrombocytopenia 28%. No new toxicity signal	[86]	Selinexor + low-intensity and targeted therapy
**Retrospective** **cohort**	Single-center retrospective series, Zhengzhou 2022–23 R/R AML (*n* = 12)	Selinexor + venetoclax + azacitidine induction	CR 41.7% (5/12), CRi 8.3% (1/12), PR 41.7% (5/12) Median time to CR 28 days (16–59); median PFS 61 days.	Predominantly hematologic toxicity; no chemotherapy-related deaths reported	[87] *	Selinexor + low-intensity and targeted therapy
**NCT02649790**	Phase 1/2, open-label Higher-risk HMA-refractory MDS with 5–19% myeloblasts (MDS/AML boundary); *n* = 20	Eltanexor 20 mg (*n* = 15) or 10 mg (*n* = 5) once daily, days 1–5 weekly of a 28-day cycle	In 15 evaluable patients: ORR 53.3%, marrow CR 46.7%. (ORR 40% in all 20 enrolled) Median OS 9.9 months.	No DLT. Nausea 45%, diarrhoea 35%, decreased appetite 35%, fatigue and neutropenia 30% each	[88]	Eltanexor
**NCT06399640**	Phase Ib R/R myelodysplastic syndrome and AML Vanderbilt-Ingram Cancer Center	Eltanexor + venetoclax	Ongoing — started August 2024; estimated primary completion February 2027	Preclinical basis: CRM1/XPO1 inhibition lowers MCL1 and synergizes with venetoclax. Splicing factor mutant AML may be more sensitive to nuclear export inhibition.	[89] ** [86]	Eltanexor
**NCT04898894** **SELCLAX**	Phase 1 with expansion cohort Pediatric and young adult (2–30 y) R/R AML or acute leukemia of ambiguous lineage	Selinexor 40 mg/m^2^ (d1, 8, 15) + venetoclax 360 mg/m^2^ (d1–21) + fludarabine/cytarabine ± G-CSF (d16–20)	Tolerable combination; early activity including primary refractory patients Estimated completion September 2027	St. Jude with Karyopharm, AbbVie and Gateway for Cancer Research	[90]	Pediatric Trial
**NCT02212561** **SELHEM Study**	Prior pediatric phase 1 studies in children with R/R AML	Selinexor With Fludarabine and Cytarabine for Treatment of Refractory or Relapsed Leukemia or Myelodysplastic Syndrome	CR/CRi in 7 of 15 evaluable (47%), 5 MRD negative, 2 MRD negative CRs after selinexor alone (day 15). MTD 55mg/m^2^ well tolerated with fludarabine/cytarabine.	Dose limiting reversible cerebellar toxicity observed at a dose level of 70 mg/m^2^.	[91]	Pediatric Trial

AE = adverse event, allo-HSCT = allogeneic hematopoietic stem cell transplantation. ANC = absolute neutrophil count. BID = twice daily. BIW = twice weekly. BSC = best supportive care. CLAG = cladribine, cytarabine, G-CSF. CR = complete remission. CRi = CR with incomplete hematologic recovery. CRp = CR with incomplete platelet recovery. DFS = disease-free survival. DLT = dose-limiting toxicity. DoR = duration of response. EFS = event-free survival. FLAG-Ida = fludarabine, cytarabine, G-CSF, idarubicin. HMA = hypomethylating agent. MDS = myelodysplastic syndrome. MEC = mitoxantrone, etoposide, cytarabine. MLFS = morphologic leukemia-free state. MRD = measurable residual disease. MTD = maximum tolerated dose. ORR = overall response rate. OS = overall survival. PFS = progression-free survival. PR = partial response. RFS = relapse-free survival. RP2D = recommended phase 2 dose. R/R = relapsed/refractory. SAE = serious adverse event. * Abstract available in English,** trial recruiting.

## Data Availability

No new data were created or analyzed in this study. Data sharing is not applicable to this article.

## Supplementary Materials

The following supporting information can be downloaded at: https://www.mdpi.com/article/10.3390/ijms27209234/s1. Refs. [133,134,135,136,137,138,139,140,141,142,143,144,145,146,147,148,149,150,151,152,153,154] are cited in the supplementary materials.
