# Supplementary material for: Hijacking the Message: Insights from Targeting mRNA Export and Translation in AML
Source: Int J Mol Sci. 2026 Oct 17;27(20):9234. doi: 10.3390/ijms27209234 (PMC13705321; doi:10.3390/ijms27209234)
Supplement: Supplementary file 1 [file ijms-27-09234-s001.zip › ijms-4605728-supplementary.pdf]

Supplemental Table S1: eIF4E targeting by ribavirin in several cancers

Citation	Cancer Type / Context	PMID
Zhou F, Culjkovic-Kraljacic B, Bach C, Feng L, Mishima Y, Borden KLB, Tenen DG. Posttranscriptional activity of eIF4E contributes to HoxA9-driven leukemogenesis. Blood Neoplasia. 2025;2(4):100132.	AML (HoxA9–eIF4E mechanism)	40979070 / PMC12447889
Assouline S, et al. Molecular targeting of the UDP-glucuronosyltransferase enzymes in high-eIF4E R/R AML: randomized phase II trial of vismodegib, ribavirin ± decitabine. Haematologica. 2023;108(11):2946-2958.	AML (clinical trial)	36951168
Attia RT, et al. Newly Synthesized Anticancer Purine Derivatives Inhibiting p-eIF4E Using Surface-Modified Lipid Nanovesicles. ACS Omega. 2023;8(41):37864-37881.	Drug design / p-eIF4E inhibitors	37867723
Zha HL, et al. Inhibition of Eukaryotic Initiating Factor eIF4E Overcomes Abemaciclib Resistance in Gastric Cancer. Curr Med Sci. 2023;43(5):927-934.	Gastric cancer	37752406
Ghram M, et al. The eukaryotic translation initiation factor eIF4E reprograms alternative splicing. EMBO J. 2023;42(7):e110496.	Mechanistic (splicing)	36843541
Burman B, et al. Pharmacodynamic and therapeutic pilot studies of single-agent ribavirin in HPV-related malignancies. Oral Oncol. 2022;128:105806.	HPV-related malignancies (clinical)	35339025
Tian Y, et al. Ribavirin inhibits the growth and ascites formation of hepatocellular carcinoma through downregulation of CARM1 and PRMT5. Toxicol Appl Pharmacol. 2022;435:115829.	Hepatocellular carcinoma	34919946
Huq S, et al. Preclinical efficacy of ribavirin in SHH and group 3 medulloblastoma. J Neurosurg Pediatr. 2021;27(4):482-488.	Medulloblastoma	33545678
Ge S, et al. Ribavirin inhibits colorectal cancer growth by downregulating PRMT5 expression and H3R8me2s/H4R3me2s accumulation. Toxicol Appl Pharmacol. 2021;415:115450.	Colorectal cancer	33577917
Huq S, et al. Repurposing the FDA-Approved Antiviral Drug Ribavirin as Targeted Therapy for Nasopharyngeal Carcinoma. Mol Cancer Ther. 2020;19(9):1797-1808.	Nasopharyngeal carcinoma	32606016
Tan H, He L, Cheng Z. Inhibition of eIF4E signaling by ribavirin selectively targets lung cancer and angiogenesis. Biochem Biophys Res Commun. 2020;529(3):519-525.	Lung cancer / angiogenesis	32736668
Xu M, et al. Suppression of oncogenic protein translation via targeting eIF4E overcomes chemo-resistance in nasopharyngeal carcinoma. Biochem Biophys Res Commun. 2019;512(4):902-907.	Nasopharyngeal carcinoma	30929914
Jin J, et al. Targeting eIF4E signaling with ribavirin as a sensitizing strategy for ovarian cancer. Biochem Biophys Res Commun. 2019;510(4):580-586.	Ovarian cancer	30739792
Urtishak KA, et al. Targeting EIF4E signaling with ribavirin in infant acute lymphoblastic leukemia. Oncogene. 2019;38(13):2241-2262.	Infant ALL	30478448
Chen J, Xu X, Chen J. Clinically relevant concentration of ribavirin selectively targets pediatric osteosarcoma and increases chemosensitivity. Biochem Biophys Res Commun. 2018;506(3):604-610.	Pediatric osteosarcoma	30454696
Xi C, et al. Inhibition of eIF4E is effective against chemo-resistance in colon and cervical cancer. Biochem Biophys Res Commun. 2018;503(4):2286-2292.	Colon & cervical cancer	29959920
Kai J, Wang Y, Xiong F, Wang S. Genetic and pharmacological inhibition of eIF4E effectively targets esophageal cancer cells and augments 5-FU's efficacy. J Thorac Dis. 2018;10(7):3983-3991.	Esophageal cancer	30174840
Cao J, Sun X, Zhang X, Chen D. Inhibition of eIF4E cooperates with chemotherapy and immunotherapy in renal cell carcinoma. Clin Transl Oncol. 2018;20(6):761-767.	Renal cell carcinoma	29086249
Casaos J, et al. Ribavirin as a potential therapeutic for atypical teratoid/rhabdoid tumors. Oncotarget. 2018;9(8):8054-8067.	Atypical teratoid/rhabdoid tumor	29487714
Tan J, Ye J, Song M, Zhou M, Hu Y. Ribavirin augments doxorubicin's efficacy in human hepatocellular carcinoma through inhibiting doxorubicin-induced eIF4E activation. J Biochem Mol Toxicol. 2018;32(1).	Hepatocellular carcinoma	29112301
Rutherford SC, et al. The eIF4E inhibitor ribavirin as a potential antilymphoma therapeutic: early clinical data. Leuk Lymphoma. 2018;59(1):256-258.	Lymphoma (clinical)	28573908
Wang G, et al. Targeting eIF4E inhibits growth, survival and angiogenesis in retinoblastoma and enhances efficacy of chemotherapy. Biomed Pharmacother. 2017;96:750-756.	Retinoblastoma	29049978

Shen X, et al. Antiviral Drug Ribavirin Targets Thyroid Cancer Cells by Inhibiting the eIF4E-β-Catenin Axis. Am J Med Sci. 2017;354(2):182-189.	Thyroid cancer	28864377
Volpin F, et al. Use of an anti-viral drug, Ribavirin, as an anti-glioblastoma therapeutic. Oncogene. 2017;36(21):3037-3047.	Glioblastoma	27941882
Kast RE, et al. The ABC7 regimen: a new approach to metastatic breast cancer using seven common drugs to inhibit EMT and augment capecitabine efficacy. Breast Cancer (Dove Med Press). 2017;9:495-514.	Metastatic breast cancer	28744157
Dai D, Chen H, Tang J, Tang Y. Inhibition of mTOR/eIF4E by ribavirin effectively enhances the effects of paclitaxel in oral tongue squamous cell carcinoma. Biochem Biophys Res Commun. 2017;482(4):1259-1264.	Oral tongue squamous cell carcinoma	27932243
Sharma S, Baksi R, Agarwal M. Repositioning of anti-viral drugs as therapy for cervical cancer. Pharmacol Rep. 2016;68(5):983-989.	Cervical cancer	27379616
Attar-Schneider O, Drucker L, Gottfried M. Migration and EMT of lung cancer can be targeted via translation initiation factors eIF4E and eIF4GI. Lab Invest. 2016;96(9):1004-1015.	Lung cancer	27501049
Yu Y, et al. eIF4E-phosphorylation-mediated Sox2 upregulation promotes pancreatic tumor cell repopulation after irradiation. Cancer Lett. 2016;375(1):31-38.	Pancreatic cancer	26945967
Volpon L, et al. Importin 8 mediates m7G cap-sensitive nuclear import of eIF4E. Proc Natl Acad Sci U S A. 2016;113(19):5263-5268.	Mechanistic (biochemistry)	27114554
Culjkovic-Kraljacic et al, Combinatiroa targeting of nuclear export and translation of RNA inhibits aggressive B-cell lymphomas, Blood, 2016; 127(7):858-68.	B-cell lymphoma	
Pettersson F, et al. Genetic and pharmacologic inhibition of eIF4E reduces breast cancer cell migration, invasion, and metastasis. Cancer Res. 2015;75(6):1102-1112.	Breast cancer	25608710
Zismanov V, et al. Multiple myeloma proteostasis can be targeted via translation initiation factor eIF4E. Int J Oncol. 2015;46(2):860-870.	Multiple myeloma	25422161
Assouline S, et al. A phase I trial of ribavirin and low-dose cytarabine for relapsed/refractory AML with elevated eIF4E. Haematologica. 2015;100(1):e7-9.	AML (clinical trial)	25425688
Zahreddine HA, et al. The sonic hedgehog factor GLI1 imparts drug resistance through inducible glucuronidation. Nature. 2014;511(7507):90-93.	AML (drug resistance mechanism)	24870236
Matassa DS, et al. Translational control in the stress adaptive response of cancer cells: a novel role for the heat shock protein TRAP1. Cell Death Dis. 2013;4(10):e851.	General cancer cell stress response	24113185
Volpon L, et al. Conformational changes induced in eIF4E by a clinically relevant inhibitor, ribavirin triphosphate. Biochem Biophys Res Commun. 2013;434(3):614-619.	Mechanistic (structural)	23583375
Bollmann F, et al. Human iNOS expression depends on CRM1- and eIF4E-mediated nucleocytoplasmic mRNA transport. Nitric Oxide. 2013;30:49-59.	Mechanistic (non-cancer, iNOS)	23471078
Kitroser E, et al. The involvement of eIF4E in extravillous trophoblast cell function. Placenta. 2012;33(9):717-724.	Mechanistic (non-cancer, placental biology)	22748716
Hayman TJ, et al. Translation initiation factor eIF4E is a target for tumor cell radiosensitization. Cancer Res. 2012;72(9):2362-2372.	General tumor radiosensitization	22397984
Kraljacic BC, et al. Inhibition of eIF4E with ribavirin cooperates with common chemotherapies in primary acute myeloid leukemia specimens. Leukemia. 2011;25(7):1197-1200.	AML	21455212
Pettersson F, et al. Ribavirin treatment effects on breast cancers overexpressing eIF4E, a biomarker with prognostic specificity for luminal B-type breast cancer. Clin Cancer Res. 2011;17(9):2874-2884.	Breast cancer	21415224
Assouline S, et al. Molecular targeting of the oncogene eIF4E in acute myeloid leukemia (AML): a proof-of-principle clinical trial with ribavirin. Blood. 2009;114(2):257-260.	AML (clinical trial)	19433856
Kentsis A, et al. Further evidence that ribavirin interacts with eIF4E. RNA. 2005;11(12):1762-1766.	Mechanistic (biochemical)	16251386
Kentsis A, Topisirovic I, Culjkovic B, Shao L, Borden KL. Ribavirin suppresses eIF4E-mediated oncogenic transformation by physical mimicry of the m7G mRNA cap. Proc Natl Acad Sci U S A. 2004;101(52):18105-18110.	Mechanistic (biochemical)<br>Head and Neck Cancers	15601771
